# “In any crisis there is an opportunity for us to learn something new”: Australian teacher experiences during COVID-19

**DOI:** 10.1007/s13384-022-00556-x

**Published:** 2022-08-20

**Authors:** Umesh Sharma, Stella Laletas, Fiona May, Christine Grove

**Affiliations:** 1 Educational Psychology and Inclusive Education, Faculty of Education, Monash University, Melbourne, Australia; 2 School of Curriculum Teaching and Inclusive Education, Faculty of Education, Monash University, Melbourne, Australia

**Keywords:** Inclusive education, Teacher resilience, Appreciative inquiry, Education and COVID-19

## Abstract

The aim of this study was to investigate the factors that supported Australian teachers to meet the needs of all learners during COVID-19 lockdowns. A qualitative design was used, and interviews were conducted with teachers who were purposely identified. The participants (*n* = 5) worked across different educational contexts ranging from primary to secondary school settings. The interview data were analysed using thematic analysis. Five themes were identified related to teachers’ intrinsic strengths: passion and commitment, a proactive and organised approach, enhanced flexibility, building relationships and access to online technology. A further three extrinsic supporting factors were identified: supportive school teams, supportive school leadership (empowerment, autonomy and time) and supportive school systems and structures. The implications of these findings are discussed and the need for sharing and applying learnings across the profession are emphasised as an opportunity to further improve the access of every learner to a high quality and inclusive education.

## Introduction

COVID-19 has disrupted education systems on a global scale, creating challenges that have never been experienced before. According to UNICEF (2020a), approximately 1.6 billion children around the world have been unable to attend school due to COVID-19 lockdowns, with schools required to make rapid adjustments in the move to online teaching and learning. For many children and young people around the world, COVID-19 has increased the educational divide. For learners with additional needs, the move to remote learning has presented further challenges, including barriers to engaging with technology, reduced access to educational supports and individualised learning interventions and a loss of social connections. In response to these challenges, many teachers have rapidly adjusted their practice and developed innovative approaches to providing inclusive education to all learners during COVID-19. Inclusion has been defined as an approach to teaching and learning that ensures all students are supported according to their individual needs to enable them to participate in and benefit from education (e.g. Ainscow & Cesar, 2006). Inclusive teachers are those who create supportive and stimulating learning environments, where students are supported to engage fully with social, academic and extra-curricular opportunities (e.g. Mastropieri & Scruggs, 2001). The purpose of this study was to explore the strengths and resilience of teachers in adopting inclusive and engaging practices in their online teaching during the COVID-19 pandemic. By sharing and applying new learnings acquired during COVID-19, the challenges experienced during this time represent an opportunity to further improve the access of every learner to a high quality and inclusive education beyond the pandemic.

### Impact of COVID-19 on Australian teachers

COVID-19 was initially identified in Australia in January 2020, with a range of measures introduced by the Australian Government to manage the pandemic including mandatory quarantine for overseas arrivals, targeted testing and contact tracing and travel restrictions (Australian Government Department of Health, 2020). By March 2020, most states and territories in Australia were in lockdown, with schools moving to home learning. By June 2020, many students across Australia returned to face-to-face learning following reductions in COVID-19 case numbers. Since this time, there has been significant variation across Australian states and territories in response to subsequent COVID-19 outbreaks, with students in the state of Victoria returning to an extended period of home learning in the second half of 2020 in response to an increase in COVID-19 cases (Victorian State Government, Department of Health and Human Services, 2020).

Consistent with the experiences of teachers all over the world (Kim & Asbury, 2020; UNICEF, 2020a), Australian teachers were required to make rapid adjustments to teaching and learning in response to the emergence of COVID-19. Prior to the pandemic, few teachers in Australian schools were using online platforms to support teaching and learning (Ziebell et al., 2020). Teachers were subsequently required to transition to remote learning platforms within a short timeframe often with little training in using such technology (Sonnemann & Goss, 2020) and to make rapid adjustments in the delivery of support to students with additional learning needs to maintain engagement and participation in school.

Early research on the effects of COVID-19 on education in Australia identified a broad range of impacts on teaching and learning. These included a lack of engagement of learners in online environments, lack of teacher knowledge about how best to use the existing technology to deliver online lessons and a lack of resources to support learners with additional needs (Flack et al., 2020). Some authors suggested new ways of working to address these challenges. These included variations to modes of working for teachers including online and offline remote teaching, increased communication with parents, a need for greater collaboration between teachers in order to work effectively in online environments and an emphasis on new approaches to monitoring student wellbeing remotely, particularly for learners at risk (Ziebell et al., 2020).

### Researchers’ positionalities

Researchers’ positionality is a critical factor that can influence how research is conducted, how data are analysed and outcomes and results are interpreted. Our team believed that COVID-19 was a difficult period for the school sector, and we were aware that the sector was under heightened stress. Rather than focusing on what was not working and what was causing stress to the schooling community, we decided to look for what was working and what we could learn from educators who, despite several challenges, were making positive contributions through their critical roles, to society. Our collective educational and professional backgrounds and experiences of working in inclusive education, educational psychology and positive psychology influenced our overall approach in this paper.

### Theoretical framework: appreciative inquiry and resilience theory

Resilience Theory provides a conceptual framework for understanding why some individuals thrive despite the risks associated with challenging circumstances (Rutter, 2006). Resilience Theory provides a strength-based approach by focussing on positive individual factors and extrinsic variables such as contextual and social factors that buffer against the impact of risk factors. Past research on teacher resilience during challenging social, health or economic circumstances (Delport et al., 2011; Wood et al., 2012) provides a useful lens for understanding teacher responses to COVID-19. Resilience has been defined as the capacity to ‘bounce back’ from adversity (Masten & Reed, 2005) with research suggesting that teachers who are resourceful and able to develop positive strategies are more likely to adapt effectively to challenging experiences (Bowles & Arnup, 2016). Teacher resilience has been described as a dynamic process involving interactions between intra-personal or intrinsic factors such as positive coping strategies, internal locus of control and optimism; and inter-personal or extrinsic factors including resources provided by families, culture and communities (Ungar, 2008).

Although COVID-19 has presented unique challenges to education systems around the world in terms of its scale and impact, we can learn about how teachers have responded to other world-wide pandemics, such as HIV/AIDS, in understanding teacher resilience. For example, resilience has been studied in the context of teachers responding to the HIV/AIDS pandemic in Lesotho (Wood et al., 2012) and also in teachers in South Africa affected by HIV/AIDS themselves (Delport et al., 2011). These studies emphasise the importance of protective resources in the form of professional support, in addition to the need for teachers themselves to draw on available individual and extrinsic resources in overcoming the challenges associated with pandemics such as HIV/AIDS (Delport et al., 2011).

This qualitative study was based on interpretive research using the strength-based ‘Appreciative Inquiry’ approach. Using a strength-based approach can serve as a key catalyst that initiates, drives and sustains positive change. Related to the literature on teacher resilience, Appreciative Inquiry aims to enable the exploration of the positive, innovative and transformational experiences of people during times of difficulty (Cooperrider, 2005; Cooperrider & Srivastva, 1987) and is based on the assumption that it is possible to bring about change in an organisation by focussing on strengths (Cooperrider et al., 2008). While it is important to acknowledge challenges, focussing solely on difficulties may miss opportunities for recognising the strengths of teachers and identifying adaptations to practice that may help progress the field of inclusive education.

Appreciative Inquiry has been applied to the field of education for many years, with applications in the areas of school improvement (e.g. Calabrese et al., 2010), whole school initiatives to improve student wellbeing (e.g. Filleul & Rowland, 2006; Waters & White, 2015), increase student voice (e.g. Bergmark & Kostenius, 2018) and initiatives to enhance student learning (Filleul & Rowland, 2006). There is also a growing body of research in the international inclusive education field, which draws on the Appreciative Inquiry approach (e.g. Calabrese et al., 2008; Iyer, 2013; Kozik et al., 2009).

The COVID-19 pandemic has led to significant disruptions to education systems around the world. However, evidence suggests that many teachers have responded with resilience (Ziebell et al., 2020), rapidly adapting their practice using innovative approaches to teaching and learning. The purpose of this study was to explore the individual capabilities, and strengths and resilience of teachers in adopting inclusive and engaging approaches in their online teaching in response to the COVID-19 pandemic, as well as the extrinsic factors which supported teachers during this time. This is important because it creates opportunities for learning and strengthening inclusive education approaches beyond the pandemic. The specific research questions guiding this investigation included:

1. How did teachers support and engage all learners during COVID-19?
2. Were there any factors extrinsic to these individuals that supported them in creating inclusive and engaging virtual classrooms for all learners?

## Method

The study used a qualitative design, including in-depth semi-structured interviews, which were analysed using inductive thematic analysis.

### Participants

Participants included five teachers from four different schools representing a range of Australian educational settings and sectors. Four teachers worked in secondary schools and one teacher in a primary school, with four teachers working in government school settings and one working in an independent school. Participants from secondary schools included teachers from a range of subject areas, including Mathematics, English, Science and Performing Arts. In addition to teaching responsibilities, all four secondary school teachers held leadership roles within their schools, with additional responsibilities for subject and year level coordination. All teachers were based in metropolitan areas, with schools servicing communities from a range of socio-economic and demographic backgrounds. Two schools were located in communities with a large number of learners from culturally and linguistically diverse backgrounds. Participating teachers included four females and one male, all with a minimum of 10 years’ experience in their role and specific experience in supporting students with additional learning needs in mainstream school settings. Participants described a range of skills and confidence in using technology to support teaching and learning prior to COVID-19, with no participants having experience with remote learning before the pandemic.

### Procedure

Nominations for teachers making a difference in the lives of learners during COVID-19 were sought from parents and carers, peers and school leaders. Nominated teachers who agreed to participate received an explanatory statement and consent form and were invited to take part in a videoconference interview with two members of the research team. Semi-structured interview schedules developed within an Appreciative Inquiry framework guided discussions with teachers, and interviews were recorded to assist with future analysis (see Appendix A).

### Data analysis

Interview responses were analysed using Braun and Clarke’s (2006) thematic analysis coding process. Information obtained across interviews were organised into codes, with conceptually related codes then combined into different themes. A series of validation meetings was held with members of the research team (US, SL, FM) to discuss codes and reach consensus on identified themes.

### Data verification

Participant validation and peer debriefing were two methods used to promote trustworthiness of the data (Burnard et al., 2008). For participant validation, the researchers invited each participant to validate, or refute, their responses throughout the interview. To elicit refinements, researchers encouraged participants to reflect and clarify their responses throughout their interview (Mertens, 2014). Allowing time for reflection provided opportunities for exploring an emotional dimension to the participant’s story (Englander, 2012).

Peer debriefings were conducted regularly to discuss emerging themes. A consensus was reached at these meetings by discussing themes in regard to transcripts and the researcher’s reflective note taking. When themes were agreed upon, the research team sought possible demographic patterns (Braun & Clarke, 2014). For instance, one consideration was whether the number of years of teaching experience influenced their experiences of using technology and online teaching. Results are reported using descriptive quotes to support the themes derived from the data.

## Results

### Teachers’ support and engagement of learners during COVID-19

Thematic analysis of teacher interviews yielded five overarching themes in regard to the ways in which teachers supported and engaged learners during COVID-19. These include passion and commitment, a proactive and organised approach, enhanced personalisation and flexibility, the importance of relationships and facilitating access to technology.

### Passion and commitment

A defining or signature personal strength reported by most participants included their passion for teaching and their commitment to supporting their learners to experience positive outcomes. Teachers described how these qualities enabled them to cope with the challenges associated with COVID-19 and to respond in flexible and innovative ways in order to best meet the needs of their students.

I love teaching…Being in the classroom is my favourite place, and while I couldn’t be “in” a classroom during remote learning, I could do everything possible to bring the classroom to the students in their own home. I’m pretty proud of what we did—we created a system that enabled students to continue to learn. (Hayley, Secondary School Teacher)

In addition, teachers drew on their experience and skills as well as demonstrating a willingness and commitment to research, apply and refine new approaches to best supporting their students' needs.

The first day of lockdown, I got up and started googling screen capture software as I knew I needed to work out how to create videos. I started playing around with different software—Loom, ScreenCastify, etc. I read blogs on making videos and worked out how to do it. (Hayley, Secondary School Teacher)

### A proactive and organised approach

Another signature strength identified by participants that enabled them to support and engage all learners during COVID-19 included their proactive and organised approach to managing the increased workload associated with moving to an online learning environment. Teachers described the importance of drawing on organisational and time management skills to enable them to rapidly adapt the curriculum and plan engaging and inclusive lessons in an online environment to ensure the needs of all students were being met, “I think time management and my organisational skills were important…For example, we had our lesson plans developed [in advance] for all the weeks [of home learning]” (Anna, Secondary School Teacher).

### Enhanced personalisation and flexibility

Individualised approaches that took into account each student’s learning needs were described as being particularly important during COVID-19. In many instances, this required teachers to apply a flexible approach to teaching and learning, making additional adjustments to the delivery and content of learning tasks.

I think we were quite flexible in our approach. The amount of curriculum and the expectations of students was reduced. For junior school, it was around 30-to-45-minute lessons and for senior school it was around one hour, and all summative assessments were eliminated to reduce that stress (Anna, Secondary School Teacher).

Teachers reported that some students with additional learning needs thrived during home learning, by allowing greater flexibility to complete learning tasks in their own time and reducing the sensory and social demands of the school environment. For other students, home learning created greater difficulties in engaging with school. Teachers acknowledged that a flexible approach responsive to the individual needs of each student was particularly important during COVID-19.

One of my students absolutely thrived [during home learning]. Working together with his mother, we supported him to take longer with tasks, take more breaks and share his ideas out loud in order to organise his thoughts before he had to apply the learning to the task at hand (Mikaela, Primary School Teacher).

Having knowledge of individual learners’ strengths and drawing on these to personalise learning was described by most teachers as being an important approach to increasing student engagement. For some students, this involved providing additional support to access the curriculum via remote learning, for others this meant modifying learning tasks so that they best aligned with students’ interests, preferences and abilities.

I had this idea that instead of homework we would do challenges of the week…So we had a Master Chef challenge, because it was very popular with some students, and the kids had to cook a meal and video themselves. We had a Lego challenge where they had to design a new building for the school…and then one student did a Survivor challenge and they invented their own Survivor task…and all the parents got involved, and they just loved it (Mikaela, Primary School Teacher).

### Building relationship with learners and their families

All teachers who participated in the study described the importance of establishing and maintaining strong connections with learners and their parents or carers during COVID-19. Teachers described the importance of investing in relationships as a means of safeguarding student wellbeing and in order to increase student engagement with school.

I made it compulsory that we would call up every single student within one or two weeks of COVID lockdown. Our aim was to have some sort of one-on-one contact with them, just to make sure that they were engaged, learning, and they were feeling supported (Tom, Secondary School Teacher).

Participating teachers also described the increased emphasis on establishing and maintaining connections with parents and careers during home learning. Teachers described this role as being important as a means of remaining connected to learners and ensuring students’ learning needs were met during home learning.

Most teachers described the importance of connecting with students using humour and a sense of fun during home learning to maintain student engagement with their learning and schools more broadly.

Staff and student wellbeing was a priority. I suggested that we do videos and I think that really boosted student and staff morale…Students and staff were really happy to see us just doing something very different, very fun (Anna, Secondary School Teacher).

Finally, teachers reported the need for them to create and nurture opportunities for learners to connect with each other during home learning to maintain social connections as well as providing peer support with learning.

I had five kids from Grade 11 who developed a gaming platform, which allowed students from every year level to join. Kids were using it as a big chat room to catch up and talk (Tom, Secondary School Teacher).

### Facilitating access to online technology

Teachers identified that for many students, the increased reliance on technology during COVID-19 contributed towards further challenges. Participants reported working together with families to ensure all learners had access to the technology they needed in order to participate in remote learning. For some families this involved access to the internet, or to devices such as laptops or tablets.

Teachers also reported that strategies such as providing students with teachers’ or education support staff phone numbers if they needed additional support, and ensuring online lessons were recorded so students could watch them in their own time and again as needed, were described as being important.

So one thing I noticed is that some learners really thrived in that environment, having the content prerecorded…it meant the kids could pause it, they could re-watch it, they could fully understand it. So [for some learners] I think their learning actually improved (Hayley, Secondary School Teacher).

### Extrinsic factors that supported teachers to create inclusive and engaging virtual classrooms for all learners

There were some common extrinsic factors evident in schools where the participating teachers were working which allowed these teachers to be so engaging, inclusive and effective during COVID-19. These factors included supportive teams, supportive leadership (empowerment, autonomy and time) and supportive school systems and structures.

### Supportive teams

Almost all teachers who participated in our study reported feeling highly supported by their colleagues and described the importance of working in effective teams in supporting them to deliver engaging and inclusive education during COVID-19. Moreover, teachers reported they were better able to meet the needs of their students and also experienced less burnout as they received support from their colleagues.

My team are just brilliant—we usually plan everything together and do all the lessons together and use all of our experience. But during home learning, that wasn't possible. So we divided and conquered. I took on maths, one of them took on specialist subjects, one of them took on English, and just having that trust in each other was so important (Mikaela, Primary School Teacher).

It appears that if participants were working in schools where there was not enough teamwork, creating an engaging online environment was more challenging. A supportive team tends to create a supportive culture.

More than ever, we had to utilise our teams. That was definitely something that improved through the COVID experience…Now that we're back at school, we've made changes to our practice based on that experience…we're sharing resources more and it's been noticeably different since coming back (Hayley, Secondary School Teacher).

### Supportive leadership

Most teachers described the importance of supportive school leadership during COVID-19 in supporting teachers and learners. A number of sub-themes were identified as being important within the theme of supportive leadership: empowerment, time and autonomy.

#### Empowerment

Teachers described the impact of feeling empowered by their school leadership team to use their initiative to identify new and innovative approaches to teaching and learning. Some participants described how their school utilised a model of ‘distributed leadership’ which built on the strengths of the broader school community and empowered teachers to draw on their intrinsic strengths in order to respond positively to the challenges associated with COVID-19.

There were no questions about what we were doing. It was always, “You've got the interests of these kids at heart, so do what you think is the right thing to do” and that's really empowering and important too. You knew that you had the support behind you and they believed in you and trusted you (Jane, Secondary School Teacher).

#### Autonomy

Participants described the benefits of being able to work autonomously, without having to seek approval from leadership regarding every decision. This freed teachers to innovate and respond rapidly to their students' needs. Teachers also described the sense of professional trust associated with being supported by leadership to work autonomously.

I never felt like I'm not allowed to make these videos or we're not allowed to do this. It was like, “Well, we're definitely meeting our baseline requirement here… what can we do over and above that?” So we had a lot of flexibility and freedom and autonomy in how we went about it, which now, looking back, I really, really appreciate (Hayley, Secondary School Teacher).

#### Time

Participants also reported the benefits associated with school leaders providing them with additional time to plan and trial new approaches to practice in order to best meet student needs during COVID-19.

### Supportive school systems and structures

Participants described the value of schools establishing systems to respond flexibly to the individual needs of learners during COVID-19, including adaptations to the school timetable, modifications to curriculum and assessment requirements and a flexible and individualised approach to student attendance that enabled teachers to meet the changing needs of students during remote learning. Teachers also described the positive impacts of school systems that enabled a more flexible approach to the delivery of the curriculum during COVID-19.

When [the school] got feedback that students were feeling that they were getting too much work, they passed that on to us and said, “Where possible, just slow down your course outlines,” because at the start we were trying to stick to our schedule...and that wasn't realistic. So we all made adjustments like that (Hayley, Secondary School Teacher).

## Discussion

The aim of this study was to understand the factors that supported teachers working in different educational contexts in Australia to meet the needs of all learners during COVID-19 and to explore the capabilities and positive potential of teachers to incorporate inclusive practices in their online teaching. The study makes a novel contribution to the literature, highlighting a number of findings with important implications for theory and practice beyond the COVID-19 pandemic.

**Fig. 1:**
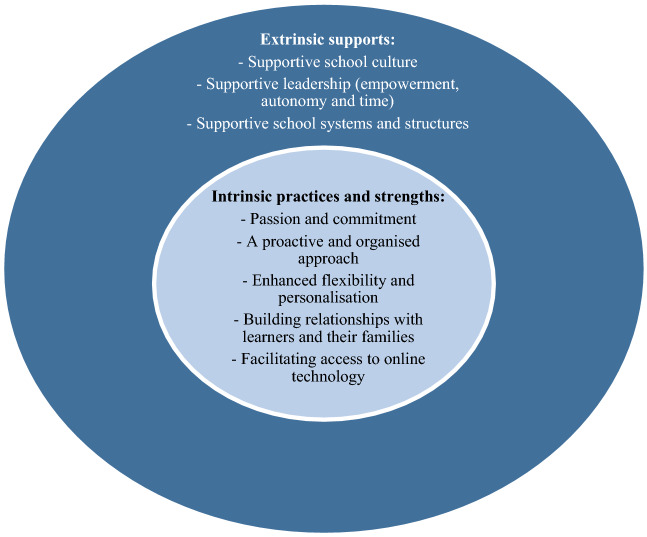
Australian teacher experiences during COVID-19: Intrinsic practices and strengths and extrinsic supporting factors. This figure includes two concentric circles, with the inner circle outlining the intrinsic practices or strengths identified by teachers, and the outer circle describing the extrinsic supports

Results of thematic analysis revealed five themes related to practices or strengths intrinsic to effective teachers and three themes related to extrinsic supporting factors that enabled teachers to provide inclusive and engaging education to support all learners during COVID-19, as illustrated in Fig. 1. Highly effective and engaging teachers, who were making a difference in the lives of all learners during COVID-19 as nominated by parents, peers and school leaders were those who were intrinsically driven and had many signature strengths. However, it is important to note that these teachers do not exist in a vacuum. They need to be fully supported to sustain their effective and inclusive practices. All our participating teachers were working in schools where various support structures existed which facilitated them be highly effective, inclusive and engaging.

The identification of a number of intrinsic practices and strengths, in addition to extrinsic supporting factors is consistent with Ungar’s (2008) conceptualisation of teacher resilience as being a dynamic process involving interactions between individual (intrinsic) and extrinsic supporting factors. Viewed through this model, teachers adapt to challenging situations by drawing on positive sources of support from the community or profession, in addition to harnessing personal strengths to enable the mobilisation of adaptive responses.

A number of intrinsic practices and strengths were identified that supported teachers to provide inclusive and engaging education to all learners during COVID-19. All teachers who participated in our study described the importance of their passion for teaching and their commitment to their students as being essential to supporting their practice during COVID-19. These findings are consistent with the growing body of literature describing the importance of teacher passion and commitment to the role (Benekos, 2016; Hammer et al., 2010; Orlando, 2013). Teachers also identified the importance of a proactive and organised approach to managing the increased demands associated with COVID-19 and in responding effectively to the needs of all learners during remote learning. Given recent findings from a review of teacher experiences of the impact of COVID-19 in Australia and New Zealand which identified that 70% of teachers reported an increase in their planning time (Flack et al., 2020) and research which identified that teachers experience increased risk of burnout due to workload demands (Heffernan et al., 2019; Skaalvik & Skaalvik, 2010), these results emphasise the importance of teacher capacity to draw on or cultivate intrinsic strengths in order to adapt to the increased challenges presented by COVID-19 and avoid negative impacts such as burnout.

Another practice identified by participants included responding with flexibility and making adaptations to the teaching and learning process in order to provide inclusive education to all students. Although this practice is a core feature of effective inclusive education (Ahsan et al., 2012; Sharma, 2018), consistent with other research (Ziebell et al., 2020), participants described the capacity to provide a flexible and individualised response to their students as being particularly important during COVID-19.

Teachers also described the importance of relationships in providing engaging and inclusive education to all learners during COVID-19. Teachers reported the quality of the relationship between students and teachers, between teachers and parents and amongst staff as being central to providing high-quality inclusive learning during COVID-19. These findings are also consistent with previous research on the experience of Australian teachers during COVID-19 (Ziebell et al., 2020) which identified increased communication with parents and greater collaboration between teachers as being critical to working effectively in online environments during COVID-19. The importance of relationships in supporting student learning during COVID-19 has been identified in studies in other countries also (e.g. Kim & Asbury, 2020) and is consistent with the broader evidence base regarding the importance of the student–teacher relationship in contributing towards improved student outcomes (e.g. Barile et al., 2012; Muller et al., 1999; O’Connor & McCartney, 2007).

Another important practice identified by participants in our study included facilitating access to online technology for all learners. Challenges associated with inequities in access to technology during COVID-19 have been widely reported (e.g. Flack et al., 2020; Sonnemann & Goss, 2020; UNICEF, 2020b). Teachers in our study, through the support of their school, were able to facilitate access to technology through the provision of devices and mobile internet dongles.

In terms of our second research question, participants identified a number of extrinsic factors that were important in supporting them to provide engaging and inclusive learning experiences during COVID-19. These included supportive teams, supportive leadership (including empowerment, autonomy and time) and supportive school systems and structures.

Consistent with the broader evidence-base regarding the role of teamwork and sense of community with teaching colleagues in supporting practice and reducing work-related stress (Hanna et al., 2019; Hargreaves, 2001; Mansfield & Gu, 2019), participants in the current study described the positive impacts associated with collegial support and effective teamwork. This support enabled teachers to work more efficiently by sharing the workload with colleagues in regard to online lesson planning and preparation, but also provided important emotional support.

Participants in the current study also identified the importance of supportive leadership in enabling them to provide engaging and inclusive education to learners during COVID-19. Teachers who worked in schools where the leadership team acknowledged the expertise of their staff and empowered teachers to use their initiative reported an increased capacity to incorporate innovations to support student engagement. Similarly, school leaders who supported their staff to work autonomously reported an increased capacity to respond rapidly to the changing needs of their students, and greater satisfaction with their work. These findings are consistent with the growing evidence-base describing the importance of supportive and distributive leadership practices in contributing towards highly effective teachers, improving student outcomes and creating inclusive school cultures (Harris et al., 2007; Heck & Hallinger, 2009; Óskarsdóttir et al., 2020; Waniganayake et al., 2015). They also underscore the importance of providing the training and support required to enable effective and inclusive school leadership in order to create and sustain inclusive school cultures (e.g. Kuyini & Desai, 2007).

Teachers involved in the current study identified a number of ways in which the inclusive practices they had adopted during COVID-19 would remain beyond the pandemic. Consistent with the research on Resilience Theory (e.g. Rutter, 2006) which suggests it is possible for individuals to thrive despite the experience of adversity, these findings are noteworthy as they demonstrate teachers’ capacity to adapt and learn from the challenges experienced during COVID-19 in order to strengthen the inclusion of all learners on the return to the classroom. This study provides important insights into the factors that enabled these peer and parent-nominated effective teachers to thrive during the pandemic, which may be helpful in informing pre-service teacher training and ongoing professional development opportunities.

## Limitations and future research

Although this study makes an important contribution to our understanding of the factors that supported Australian teachers to provide engaging and inclusive education during COVID-19, our findings must be considered within the context of the study’s limitations. Although some participants were nominated by parents or their school’s leadership team, two participants were self-nominated, which means the experiences reported in the current study may represent the views of teachers who had more positive experiences during COVID-19, due to the nature of their individual contexts or the type of support they received in their schools. The small sample also limits the study’s capacity to generalise findings. The results, therefore, need to be considered with the caveat that they may not represent the range of views and experiences held by the broader population of Australian teachers. Similarly, the study’s focus on identifying strengths may have precluded a more detailed investigation of barriers to teachers providing inclusive and engaging education to learners during COVID-19. However, the goals of this study were to provide a more in-depth exploration of strengths and supporting factors to enable this knowledge to be shared across the profession and to further strengthen opportunities for inclusive education for all learners.

## Conclusions

Despite the many challenges experienced during COVID-19, teachers in our study responded with creativity, flexibility and innovation to support the inclusion of all learners. Consistent with models of teacher resilience (Ungar, 2008), this research identified a number of inter-connected intrinsic strengths, including passion and commitment, a proactive and organised approach, enhanced flexibility, building relationships and access to online technology, and extrinsic supporting factors including supportive school teams, supportive school leadership and supportive school systems that enabled teachers to provide engaging and inclusive learning experiences for all students. Of particular note, teachers described how many of the innovations adopted during COVID-19 have continued to be applied on return to the classroom, resulting in further benefits for students. Given the identified risks of students being left behind by education systems due to the challenges presented by COVID-19, it is important that the strengths, practices and supporting factors that enable teachers to provide a high-quality education to all learners are identified and shared across the profession. Now more than ever, it is essential that we listen and learn from the experiences of teachers and acknowledge the outstanding contributions they are making in supporting the engagement and inclusion of all learners.

## Appendix A: Semi-structured interview schedule for teachers

1. Please share your story of teaching during the COVID-19 pandemic.
2. What were some of the most significant challenges you faced? Is there any particular challenge that stands out for you? How did you address the challenge?
3. Is there any particular story/event that stands out for you which clearly shows that you made a difference for a vulnerable learner/family—and why it stands out for you. Please share the story. What happened? Where? When? How did you feel? How did you overcome challenges? What contributions?
4. Now, from this story and without being too humble about it, what does this story say about you–your three most important or signature strengths? Are those strengths intrinsic to you or was there something extrinsic to you?
